# Selecting Cooking Methods to Decrease Persistent Organic Pollutant Concentrations in Food of Animal Origin Using a Consensus Decision-Making Model

**DOI:** 10.3390/ijerph14020187

**Published:** 2017-02-14

**Authors:** Xiao Tan, Zaiwu Gong, Minji Huang, Zhou-Jing Wang

**Affiliations:** 1 School of Economics and Management, Nanjing University of Information Science and Technology, Nanjing 210044, China; ctanxiao@163.com (X.T.); zwgong26@163.com (Z.G.); 2 Collaborative Innovation Center on Forecast and Evaluation of Meteorological Disasters, Nanjing 210044, China; 3 Fujian Education Examinations Authority, 59 Beihuanzhong Road, Fuzhou 350003, Fujian, China; huangminji331@hotmail.com; 4 Department of Automation, Xiamen University, Xiamen 361005, China; 5 School of Information, Zhejiang University of Finance & Economics, Hangzhou 310018, China

**Keywords:** POPs, animal source food, interval preference relation, consensus, consistency, social choice principles

## Abstract

Persistent organic pollutants (POPs) pose serious threats to human health. Increasing attention has been paid to POPs to protect the environment and prevent disease. Humans are exposed to POPs through diet (the major route), inhaling air and dust and skin contact. POPs are very lipophilic and hydrophobic, meaning that they accumulate in fatty tissues in animals and can biomagnify. Humans can therefore be exposed to relatively high POP concentrations in food of animal origin. Cooking animal products can decrease the POP contents, and different cooking methods achieve different reduction rates. Here, a consensus decision-making model with interval preference relations is used to prioritize cooking methods for specific animal products in terms of reducing POP concentrations. Two consistency mathematical expressions (*I*-consistency and II-consistency) are defined, then the ideal interval preference relations are determined for the cooking methods with respect to different social choice principles. The objective is to minimize disparities between individual judgments and the ideal consensus judgment. Consistency is used as a constraint to determine the rationality of the consistency definitions. A numerical example indicated that baking is the best cooking method for decreasing POP concentrations in grass carp. The *I*-consistency results were more acceptable than the II-consistency results.

## 1. Introduction

Persistent organic pollutants (POPs) are a serious environmental problem. POPs are toxic, can be transported long distances in the environment and can accumulate in animals and humans and biomagnify through the food chain. POPs are persistent in various environmental media, including soil, air, water and organisms. Many scholars have studied the harm to the human body resulting from the exposure of POPs by analyzing experimental data [1,2]. The main sources of POPs ingested by humans are foods of animal origin that are included in the daily diet [3]. It has been found in many studies that conventional cooking methods, such as frying, baking, poaching and pressure cooking, can affect the POP contents and distributions in foods of animal origin [4], and experiments have verified that different cooking methods affect the contents and distributions to different degrees [5,6]. Studying the effects of different cooking methods on POP concentrations in foods of animal origin is difficult. This is because different foods are eaten; animals used to provide food live under different environmental conditions; and different cooking environments and methods (including the use of oils and seasonings containing volatile chlorinated organic compounds) are used in different areas. It is impractical to perform experiments to test POP concentrations in foods of animal origin under every possible combination of these variables. In the study presented here, a consensus model using two consistency definitions to aggregate the judgments of experts is proposed. Experts can provide more reasonable judgments than non-experts about the effects of variations in the factors summarized above. The judgments used in the model are in the forms of interval values. Different experts have different backgrounds and specialties, so their preferences need to be determined from a number of perspectives. Using different social choice function (SCF) decision-making rules to determine the optimal cooking method to decrease POP concentrations in food of animal origin is therefore appropriate. The advantages of this study are that aiming at the complexity of POPs and experiments, we supply a reasonable and simple selection method based on a relative technique (experts with experimental experience); this method is a more comprehensive subjective judgment selection system combining the social principles (representing choice characteristics) and consistency conditions (representing the logic of experts’ opinions); the shortcoming is exposed simultaneously: although the judgment of experts is established on the basis of experience, it is also subjective; obviously, the experimental method is more reliable; however, with the limited conditions, this can yet be regarded as a kind of quick and convenient method.

Individual preferences can be described using a series of uncertain fuzzy numbers, such as an interval number, an intuitionistic fuzzy number and a hesitant fuzzy number, to represent uncertain fuzzy preference relations, such as the interval fuzzy preference relation, the intuitionistic fuzzy preference relation and the hesitant fuzzy preference relation, respectively, to express the degree to which one alternative is preferred over another. The study presented here is focused on how the individuals’ uncertain preference information can be fully integrated into the consensus preference relations based on SCF to give a satisfactory group decision-making solution. Consensus decision-making (CDM) with preferences is used to investigate consistency and reach consensus. Consistency in individual preferences is the basis of CDM, and this reflects the logic of the decision-makers (DMs). Consensus is the combined view of a decision-making problem, and the CDM rules are essentially reflected by social choices and group preferences. The roles and meanings of consistency, consensus and rules in CDM in the model are described below.

(1) Consistency: The consistency of an individual judgment is used to determine whether the judgments of the DMs are logical. The aims of the study include evaluating the ability of the DMs to make judgments and prioritizing the alternative decisions. The use of interval fuzzy preference, triangular fuzzy preference or intuitionistic fuzzy preference by the DMs depends on the specialty and knowledge of each DM supplying judgments about the decision that should be made. Every type of preference relation has a consistency definition because of discrepancies in the mathematical structure of the model. This is also related to certain logical formulae. Similar studies have been focused on combining consistency and preference relations, for example interval fuzzy preference relations [7,8], triangular fuzzy reciprocal preference relations [9,10] and intuitionistic fuzzy preference relations [11,12]. Liu et al. [9] and Wang et al. [13] defined additive consistent fuzzy preference relations. Wang et al. [10] defined multiplicative consistency. Xu et al. [8] defined both additive and multiplicative consistency. Hu et al. [14] improved the consistency definitions of interval fuzzy preference relations using previously published information [8]. Other researchers have proposed novel models and compared them with previously available methods in terms of consistency [13,14,15].

(2) Consensus: The DMs reach consensus through mutual cooperation and compromise. The objective of CDM is to determine the priority (weight) of each alternative, to determine the degree of consensus, to identify disagreements and to control the convergence of views. In terms of the protocol involved, in 1978, Bezdek et al. [16] stated that “consensus means a complete and consistent protocol”. From the psychological preference point of view, in 1988, Orive [17] stated that consensus means the opinions of most of the DMs. From the modeling perspective, Chiclana et al. [18] defined “consensus” as the general or most popular opinion and used a similarity function to estimate the distances between the opinions of different specialists.

Consensus with uncertain preference relations requires not only the consistency of the opinions of an individual DM to be determined, but also differences between the opinions of different DMs to be minimized. Many researchers have used a distance and similarity function to measure the degree of consensus. For example, Zhang [19] and González-Pachón and Romero [20] established a consensus model based on a distance formula. Chu et al. [15] and Wu et al. [21] simultaneously addressed the functions of consistency and consensus in group decision-making.

(3) Rule of CDM: The aim of CDM is to coordinate all of the individuals and to combine the opinions given, balancing the behaviors of different DMs with different backgrounds in studying complex and variable environments. CDM can help the DMs harmoniously reach a decision. Different distance functions (such as minimization of the consensus deviation or minimization of the maximum deviation) are used to achieve this. In reality, some SCFs can be identified using these functions [20]. The aim of Benthamism [22] (the freedom principle) is to elaborate on the opinion of each individual to maximize the total value. In Rawlsianism [23] (the fraternity principle), importance is attached to the DM with the worst qualifications. Additionally, equal consideration is given to each DM in Marxism (the equality principle).

Previous studies of consensus with uncertain preference relations have mostly only taken consistency conditions or the SCFs into consideration. In the study presented here, uncertain consensus models combining consistency and the social principles (the freedom, fraternity, equality and mixed principles) are developed. Fuzzy relations, such as the intuitionistic fuzzy preference relation, hesitant fuzzy preference relation, triangular fuzzy preference relation and linguistic fuzzy preference relation, have similar mathematical structures to the interval fuzzy preference relation, so consensus models with interval fuzzy preference relations will be explored. The goal is to minimize the distance between the individual judgments and the ideal consensus judgment, and consistency is the constraint.

Two interval fuzzy preference relations with *I*-consistency and II-consistency are defined in Section 2. The decision rules for the ideal interval fuzzy preference relations are analyzed in Section 3. The goal programming models with *I*-consistency and II-consistency are presented in Section 4 and Section 5, respectively, for different social choice modes. The proposed method is applied to the selection of cooking methods in Section 6 to demonstrate that the model can be used in the health field. Concluding remarks are offered in Section 7.

## 2. Definition of Two Consistent Interval Fuzzy Preference Relations

For simplicity, we use N={1,2,…,n} and M={1,2,…,m}.

In decision-making, pairwise comparisons (Saaty, 1977, 1980) are often used by DMs to compare a set of alternative decisions with respect to a specified criterion. For a set of alternatives $X=\{x_{1},x_{2},\ldots ,x_{n}\}$, the preference information for pairwise comparisons with respect to a single criterion is represented numerically using the positive interval matrix $R={\left(r_{ij}\right)}_{n\times n}$, which can be between 0.1 and 0.9, where entry $r_{ij}\in \left[0.1,\;0.9\right]$ is an estimate of the degree or intensity of the preference for alternative $x_{i}$ over $x_{j}$. In particular, $r_{ij}=\left[0.5,\;0.5\right]$ indicates that $x_{i}$ and $x_{j}$ are of equal preference; $r_{ij}>\left[0.5,\;0.5\right]$ indicates that $x_{i}$ is preferred to $x_{j}$; and $r_{ij}<\left[0.5,\;0.5\right]$ indicates that $x_{j}$ is preferred to $x_{i}$.

**Definition** **1.** *[24] The interval matrix* $R={\left(r_{ij}\right)}_{n\times n}$ *is called the interval fuzzy preference relation if:*

(1) $$\begin{array}{ccc} r_{ij} & = & \left[0.5,0.5\right] \end{array}$$

(2) $$\begin{array}{ccc} r_{ijl}+r_{jiu} & = & r_{iju}+r_{jil}=1, \end{array}$$

*where* $r_{ij}=\left[r_{ijl},r_{iju}\right]$, i,j∈N.

The *I*-consistency and II-consistency expressions are defined in terms of the structural relationships between the matrix elements and between the weights, respectively.

**Definition** **2.** *For all* i,j,s∈N, i≠j≠s*, if* $r_{ij}$, $r_{ij}\in \left[r_{ijl},\;r_{iju}\right]$ *such that*

(3) $$r_{ij}+r_{js}=r_{is}+0.5,$$

*then R is called the consistent interval fuzzy preference relation with I-consistency.*

Note: The definitionof *I*-consistency in interval fuzzy preference relations is derived from the consistency definition for fuzzy preference relations [25,26].

**Definition** **3.** *[27] If* $\tilde{\Omega }={\left({\tilde{\omega }}_{1},{\tilde{\omega }}_{2},\ldots ,{\tilde{\omega }}_{n}\right)}^{T}$ *is the priority vector for the fuzzy preference relations* $\tilde{R}={\left({\tilde{r}}_{ij}\right)}_{n\times n}$*, then:*

(4) $${\tilde{r}}_{ij}=\frac{{\tilde{\omega }}_{i}}{{\tilde{\omega }}_{i}+{\tilde{\omega }}_{j}},i,j\in N.$$

In Section 4, we will define an ideal fuzzy preference relation with *I*-consistency, for which the priority vector is obtained from this definition.

**Definition** **4.** *[12] The interval fuzzy preference relation* $R={\left(r_{ij}\right)}_{n\times n}$ *is called the consistent interval fuzzy preference relation with* II*-consistency if the priority vector* $\Omega ={\left(\omega_{1},\omega_{2},\ldots ,\omega_{n}\right)}^{T}$ *of R satisfies:*

(5) $$r_{ijl}\leq \frac{\omega_{i}}{\omega_{i}+\omega_{j}}\leq r_{iju},i,j\in N,$$

*where* $\sum_{i=1}^{n}\omega_{i}=1,\;0\leq \omega_{i}\leq 1$.

*I*-consistency emphasizes the logical relationship between the matrix elements, and II-consistency emphasizes the relationship between the elements and weights.

## 3. Decision Rules for Ideal Interval Fuzzy Preference Relations

Assuming that there are *m* DMs $d_{1},d_{2},\ldots$, and $d_{m}$ in the CDM, the corresponding weights will be $\hat{\omega_{1}},\hat{\omega_{2}},\ldots$, $\hat{\omega_{m}}$, and the interval fuzzy preference relations will be:

$$\begin{array}{c} \begin{array}{c} R^{k}={\left(r_{ij}^{k}\right)}_{n\times n}=\left(\begin{array}{cccc} r_{11}^{k} & r_{12}^{k} & \cdots & r_{1n}^{k}\\ r_{21}^{k} & r_{22}^{k} & \cdots & r_{2n}^{k}\\ \vdots & \vdots & \ddots & \vdots \\ r_{n1}^{k} & r_{n2}^{k} & \cdots & r_{nn}^{k} \end{array}\right) \end{array}, \end{array}$$

where $r_{ij}^{k}=\left[r_{ijl}^{k},r_{iju}^{k}\right]$, satisfying $r_{ijl}^{k}+r_{jiu}^{k}=1$, $r_{iju}^{k}+r_{jil}^{k}=1$, i,j∈N,k∈M.

We assume that there is an ideal DM $d^{*}$ and define the interval fuzzy preference relation for $d^{*}$ as:

$$\begin{array}{c} \begin{array}{c} R^{*}=\left(\begin{array}{cccc} r_{11}^{*} & r_{12}^{*} & \cdots & r_{1n}^{*}\\ r_{21}^{*} & r_{22}^{*} & \cdots & r_{2n}^{*}\\ \vdots & \vdots & \ddots & \vdots \\ r_{n1}^{*} & r_{n2}^{*} & \cdots & r_{nn}^{*} \end{array}\right) \end{array}, \end{array}$$

where $0\leq r_{ij}^{*}\leq 1$. Using the consistency preference relations described in Section 2, the ideal DM preference relations can be presented in two forms.

- Form I: Element $r_{ij}^{*}$ is a crisp number, i.e., $r_{ij}^{*}\in \left[0,1\right]$.
- Form II: Element $r_{ij}^{*}$ is an interval number, i.e., $r_{ij}^{*}\subseteq \left[0,1\right]$.

As the research in [20], let us consider the distance function:

(6) $$U=[\sum_{k=1}^{m}\sum_{i=1}^{n}\sum_{j=1}^{n}\hat{w_{k}^{p}}|r_{ij}^{k}-r_{ij}^{*}{{|}^{p}]}^{\frac{1}{p}}.$$

This indicates the weighted average of the deviation between the ideal judgment value $r_{ij}^{*}$ and the judgments of *m* DMs $r_{ij}^{k}$. *p* (p∈[1,∞]) represents different consensus choice rules.

When p=1, Formula (6) is for consensus with the freedom principle. This reflects Bentham utilitarianism [18] and identifies optimal total social welfare taking the benefit of each individual into consideration. This system maximizes the well-being of all, rather than of a particular individual.

When p=∞, Formula (6) is for consensus with the fraternity principle. This reflects Rawls’ theory of justice [19], in which it is advocated that all members of society should be respected, rather than weak members being ignored because of the relative differences between individuals. This system emphasizes maximizing the welfare of the least benefited individual.

Consensus with the equality principle can be achieved on the basis of the freedom principle. This reflects Marxist fairness, which requires social wealth to be distributed equally and fully guarantees equality.

Consensus with mixed principles is a more general (compound) social choice mode. This is achieved by linearly combining the three specific ethical rules, simultaneously considering the freedom, fraternity and equality principles.

We will now assess the ideal fuzzy preference relations $R^{*}$ with the two consistency conditions under the freedom, fraternity, equality and mixed principles.

## 4. Construction of Ideal Fuzzy Preference Relations with I-Consistency Using Different Social Choice Modes

Considering *I*-consistency, although the values for the judgments made by the DMs are intervals, the ideal values are crisp numbers, which allow the weight values for these preference relations (using Definition 3) to be determined.

### 4.1. Construction of Ideal Fuzzy Preference Relations with I-Consistency Using the Freedom Principle

When p=1, *U* is the weighted average deviation between the ideal judgment $r_{ij}^{*}$ and judgments $r_{ij}^{k}$ made by *m* DMs. *U* is the sum of the consensus deviations and should be as small as possible. The optimal ideal preference relation using the freedom principle is:

(7) $$\begin{array}{c} \begin{array}{cc} Min\;\sum_{k=1}^{m}\sum_{i=1}^{n}\sum_{j=1}^{n}\hat{w_{k}}\left(n_{ij}^{k}+p_{ij}^{k}\right)+\sum_{i=1}^{n}\sum_{i=1}^{n}\left(d_{il}^{-}+d_{il}^{+}\right) & \\ s.t.\left\{\begin{array}{cc} r_{ij}^{k}-r_{ij}^{*}=n_{ij}^{k}-p_{ij}^{k} & \left(7a\right)\\ r_{ij}^{k}=r_{ijl}^{k}+\alpha_{ij}^{k}\left(r_{iju}^{k}-r_{ijl}^{k}\right) & \left(7b\right)\\ r_{ij}^{*}+r_{jl}^{*}-\left(r_{il}^{*}+0.5\right)+d_{il}^{-}-d_{il}^{+}=0 & \left(7c\right)\\ 0\leq r_{ij}^{*}\leq 1,0\leq \alpha_{ij}^{k}\leq 1,i,j\in N,k\in M & \left(7d\right) \end{array}\right. \end{array} \end{array}$$

where $n_{ij}^{k}+p_{ij}^{k}=\left|r_{ij}^{k}-r_{ij}^{*}\right|$, $n_{ij}^{k}-p_{ij}^{k}=r_{ij}^{k}-r_{ij}^{*}$; $r_{ij}^{k}=\left[r_{ijl}^{k},\;r_{iju}^{k}\right]=r_{ijl}^{k}+\alpha_{ij}^{k}\left(r_{iju}^{k}-r_{ijl}^{k}\right)$, $0\leq \alpha_{ij}^{k}\leq 1$. Formula (7c) is the consistency constraint, which indicates that the ideal preference relation satisfies consistency Condition (3) as much as possible, where $d_{il}^{+}$ and $d_{il}^{-}$ are the positive and negative deviations, respectively, of $\left(r_{ij}^{*}+r_{jl}^{*}\right)$ between $\left(r_{il}^{*}+0.5\right)$. Clearly, $\left(d_{il}^{+}+d_{il}^{-}\right)$ in the objective function should be as small as possible.

The objective function of Model (7) indicates that the ideal judgment $r_{ij}^{*}$ is for the minimum deviations in consensus and consistency. For the freedom principle, the optimal (ideal) consensus with appropriate consistency is achieved using Model (7).

### 4.2. Construction of Ideal Fuzzy Preference Relations with I-Consistency Using the Fraternity Principle

When p=∞, *U* is the maximum deviation between the ideal judgment $r_{ij}^{*}$ and judgments $r_{ij}^{k}$ made by *m* DMs, where $D=Max_{k,i,j}\hat{\omega_{i}}\left|r_{ij}^{k}-r_{ij}^{*}\right|$ represents the maximum consensus deviation between the ideal DM and *m* DMs. Under these circumstances, the smaller the deviations are for all of the DMs the better, i.e., the value should be as small as possible, and MinD is the minimized maximum deviation.

The optimal ideal preference relation using the fraternity principle is:

(8) $$\begin{array}{c} \begin{array}{cc} Min\;D+\sum_{i=1}^{n}\sum_{i=1}^{n}\left(d_{il}^{-}+d_{il}^{+}\right) & \\ s.t.\left\{\begin{array}{cc} r_{ij}^{k}-r_{ij}^{*}=n_{ij}^{k}-p_{ij}^{k} & \left(8a\right)\\ r_{ij}^{k}=r_{ijl}^{k}+\alpha_{ij}^{k}\left(r_{iju}^{k}-r_{ijl}^{k}\right) & \left(8b\right)\\ r_{ij}^{*}+r_{jl}^{*}-\left(r_{il}^{*}+0.5\right)+d_{il}^{-}-d_{il}^{+}=0 & \left(8c\right)\\ \hat{w_{k}}\sum_{i=1}^{n}\sum_{j=1}^{n}\left(n_{ij}^{k}+p_{ij}^{k}\right)-D\leq 0 & \left(8d\right)\\ 0\leq r_{ij}^{*}\leq 1,0\leq \alpha_{ij}^{k}\leq 1,i,j\in N,k\in M & \left(8e\right) \end{array}\right. \end{array} \end{array}$$

The parameters used in Model (8) have the same definitions as those used in Model (7). Formula (8d) indicates that all of the consensus deviations are no more than the maximum *D* value.

The objective function in Model (8) indicates that the ideal judgment $r_{ij}^{*}$ is for the minimum deviation in the maximum consensus and consistency. For the fraternity principle, the optimal consensus (ideal) judgment with appropriate consistency is achieved using Model (8).

### 4.3. Construction of Ideal Fuzzy Preference Relations with I-Consistency Using the Equality Principle

If we let:

(9) $$\hat{\omega_{1}}|r_{ij}^{1}-r_{ij}^{*}|=\hat{\omega_{2}}|r_{ij}^{2}-r_{ij}^{*}|=\ldots =\hat{\omega_{m}}\left|r_{ij}^{m}-r_{ij}^{*}\right|,$$

then Equation (9) indicates that the weights for the deviations between the ideal DM and each DM will be equal in the CDM system. The system function has the form shown below to ensure that the equations shown in Formula (9) are valid.

(10) $$\begin{array}{c} \hat{\omega_{k}}\left(n_{ij}^{k}+p_{ij}^{k}\right)=\hat{\omega_{t}}\left(n_{ij}^{t}+p_{ij}^{t}\right)\\ \;k=1,\ldots ,m-1,\;t=k+1,\ldots ,m. \end{array}$$

The optimal ideal preference relations for the equality principle are constructed as shown below.

(11) $$\begin{array}{c} \begin{array}{cc} Min\;\sum_{k=1}^{m-1}\sum_{t=k+1}^{m}\left(\eta_{ij}^{kt}+\rho_{ij}^{kt}\right)+\sum_{i=1}^{n}\sum_{i=1}^{n}\left(d_{il}^{-}+d_{il}^{+}\right) & \\ s.t.\left\{\begin{array}{cc} r_{ij}^{k}-r_{ij}^{*}=n_{ij}^{k}-p_{ij}^{k} & \left(11a\right)\\ r_{ij}^{k}=r_{ijl}^{k}+\alpha_{ij}^{k}\left(r_{iju}^{k}-r_{ijl}^{k}\right) & \left(11b\right)\\ r_{ij}^{*}+r_{jl}^{*}-\left(r_{il}^{*}+0.5\right)+d_{il}^{-}-d_{il}^{+}=0 & \left(11c\right)\\ \hat{\omega_{k}}\left(n_{ij}^{k}+p_{ij}^{k}\right)-\hat{\omega_{t}}\left(n_{ij}^{t}+p_{ij}^{t}\right)+\eta_{ij}^{kt}-\rho_{ij}^{kt}=0,\\ k=1,...,m-1,t=k+1,...,m & \left(11d\right)\\ 0\leq r_{ij}^{*}\leq 1,0\leq \alpha_{ij}^{k}\leq 1,i,j\in N,k\in M & \left(11e\right) \end{array}\right. \end{array} \end{array}$$

The parameters used in Model (11) have the same definitions as the parameters used in Model (7). Formula (11d) indicates that the weights for the deviations between the ideal DM and each DM will be as equal as possible. $\eta_{ij}^{kt}$ and $\rho_{ij}^{kt}$ are the positive and negative deviations, respectively, between $\hat{\omega_{k}}\left(n_{ij}^{k}+p_{ij}^{k}\right)$ and $\hat{\omega_{t}}\left(n_{ij}^{t}+p_{ij}^{t}\right)$. Clearly, $\left(\eta_{ij}^{kt}+\rho_{ij}^{kt}\right)$ in the objective function should be as small as possible.

The objective function of Model (11) indicates that the model can ensure that the weights of the deviations between the ideal judgment $r_{ij}^{*}$ and the judgments made by the DMs are as equal as possible and that deviations in the consistency are minimized. For the equality principle, the optimal consensus (ideal) judgment with appropriate consistency is achieved using Model (11).

### 4.4. Construction of Ideal Fuzzy Preference Relations with I-Consistency Using the Mixed Principles

The ideal fuzzy preference relations using the three individual principles (implying that there are different social choice patterns) were established in Section 4.1, Section 4.2 and Section 4.3. Linearly combining the three ethical principle Models ((7), (8) and (11)) gives a more general social choice pattern. Weights $\lambda_{i}$, $0\leq \lambda_{i}\leq 1$, i=1,2. for the different choice patterns are introduced to allow a consensus model, Model (12), based on mixed social choices to be built. In Model (12), $\lambda_{1}$, $\lambda_{2}$ and $\left(1-\lambda_{1}-\lambda_{2}\right)$, which are determined by the decision-makers, are the weights for the freedom, fraternity and equality principles, respectively. The parameters used in Model (12) have the same definitions as the parameters used in the three models described earlier.

(12) $$\begin{array}{c} \begin{array}{cc} Min\; & \lambda_{1}D+\lambda_{2}\sum_{k=1}^{m}\sum_{i=1}^{n}\sum_{j=1}^{n}\hat{\omega_{k}}\left(n_{ij}^{k}+p_{ij}^{k}\right)+\left(1-\lambda_{1}-\lambda_{2}\right)\sum_{k=1}^{m-1}\sum_{t=k+1}^{m}\left(\eta_{ij}^{kt}+\rho_{ij}^{kt}\right)+\sum_{i=1}^{n}\sum_{i=1}^{n}\left(d_{il}^{-}-d_{il}^{+}\right)\\ & s.t.\left\{\begin{array}{cc} r_{ij}^{k}-r_{ij}^{*}=n_{ij}^{k}-p_{ij}^{k} & \left(12a\right)\\ r_{ij}^{k}=r_{ijl}^{k}+\alpha_{ij}^{k}\left(r_{iju}^{k}-r_{ijl}^{k}\right) & \left(12b\right)\\ r_{ij}^{*}+r_{jl}^{*}-\left(r_{il}^{*}+0.5\right)+d_{il}^{-}-d_{il}^{+}=0 & \left(12c\right)\\ \hat{\omega_{k}}\sum_{i=1}^{n}\sum_{j=1}^{n}\left(n_{ij}^{k}+p_{ij}^{k}\right)-D\leq 0 & \left(12d\right)\\ \hat{\omega_{k}}\left(n_{ij}^{k}+p_{ij}^{k}\right)-\hat{\omega_{t}}\left(n_{ij}^{t}+p_{ij}^{t}\right)\\ +\eta_{ij}^{kt}-\rho_{ij}^{kt}=0, & \left(12e\right)\\ k=1,...,m-1,t=k+1,...,m\\ 0\leq r_{ij}^{*}\leq 1,0\leq \alpha_{ij}^{k}\leq 1,\\ i,j\in N,k\in M & \left(12f\right) \end{array}\right. \end{array} \end{array}$$

The objective function of Model (12) indicates that the sum of the products of all of the deviations and corresponding weights using different social choice patterns are minimized, and the deviation in the consistency is also minimized. For the mixed principle, the optimal (ideal) consensus with appropriate consistency is achieved using Model (12).

The consensus model described above is relatively simply solved because the procedure turns nonlinear functions into linear models.

## 5. Construction of Ideal Fuzzy Preference Relations with II-Consistency Using Different Social Choice Modes

Using II-consistency, the ideal DM judgment values and the values for the judgments made by the DMs are intervals $r_{ij}^{*}=\left[r_{ijl}^{*},r_{iju}^{*}\right]$, and $r_{ij}^{k}=\left[r_{ijl}^{k},r_{iju}^{k}\right]$, respectively. Similar construction principles to those used for *I*-consistency are used, and the ideal fuzzy preference relations with II-consistency using different social principles are described below

### 5.1. Construction of Ideal Fuzzy Preference Relations with II-Consistency Using the Freedom Principle

Similar to Section 4.1, when p=1, *U* is the weighted average of the consensus deviation between the ideal DM $d^{*}$ and mDMs. Clearly, the value should be as small as possible. The optimal ideal preference relation using the freedom principle is shown below.

(13) $$\begin{array}{c} \begin{array}{ccc} \;Min\; & & \sum_{k=1}^{m}\sum_{i=1}^{n}\sum_{j=1}^{n}\hat{\omega_{k}}\left(n_{ijl}^{k}+p_{ijl}^{k}\right)+\sum_{k=1}^{m}\sum_{i=1}^{n}\sum_{j=1}^{n}\hat{\omega_{k}}\left(n_{iju}^{k}+p_{iju}^{k}\right)\\ & & s.t.\left\{\begin{array}{cc} r_{ijl}^{k}-r_{ijl}^{*}=n_{ijl}^{k}-p_{ijl}^{k} & \left(13a\right)\\ r_{iju}^{k}-r_{iju}^{*}=n_{iju}^{k}-p_{iju}^{k} & \left(13b\right)\\ r_{ijl}^{*}\leq \frac{\omega_{i}}{\omega_{i}+\omega_{j}}\leq r_{iju}^{*} & \left(13c\right)\\ \sum_{i=1}^{n}\omega_{i}=1 & \left(13d\right)\\ i,j\in N,k\in M & \left(13e\right) \end{array}\right. \end{array} \end{array}$$

Model (13) is nonlinear, but it would be convenient to convert it into a linear model to allow a solution to be determined. We will multiply both sides of the constraint Equations (13a) and (13b) by $\omega_{i}$ and $\omega_{j}$ and multiply both sides of Inequation (13c) by ($\omega_{i}+\omega_{j}$). Model (13) then has the form shown below.

(14) $$\begin{array}{c} \begin{array}{ccc} \;Min\; & & \sum_{k=1}^{m}\sum_{i=1}^{n}\sum_{j=1}^{n}\hat{\omega_{k}}\left(n_{ijl}^{k}+p_{ijl}^{k}\right)+\sum_{k=1}^{m}\sum_{i=1}^{n}\sum_{j=1}^{n}\hat{\omega_{k}}\left(n_{iju}^{k}+p_{iju}^{k}\right)\\ & & s.t.\left\{\begin{array}{cc} r_{ijl}^{k}\omega_{i}-r_{ijl}^{*}\omega_{i}=n_{ijl}^{k}\omega_{i}-p_{ijl}^{k}\omega_{i} & \left(14a\right)\\ r_{ijl}^{k}\omega_{j}-r_{ijl}^{*}\omega_{j}=n_{ijl}^{k}\omega_{j}-p_{ijl}^{k}\omega_{j} & \left(14b\right)\\ r_{iju}^{k}\omega_{i}-r_{iju}^{*}\omega_{i}=n_{iju}^{k}\omega_{i}-p_{iju}^{k}\omega_{i} & \left(14c\right)\\ r_{iju}^{k}\omega_{j}-r_{iju}^{*}\omega_{j}=n_{iju}^{k}\omega_{j}-p_{iju}^{k}\omega_{j} & \left(14d\right)\\ r_{ijl}^{k}\omega_{i}+r_{ijl}^{k}\omega_{j}\leq \omega_{i}\leq r_{iju}^{k}\omega_{i}+r_{iju}^{*}\omega_{j} & \left(14e\right)\\ \sum_{i=1}^{n}\omega_{i}=1 & \left(14f\right)\\ i,j\in N,k\in M & \left(14g\right) \end{array}\right. \end{array} \end{array}$$

We let:

$$\begin{array}{c} n_{ijl}^{k}\omega_{i}=\bar{n_{ijl}^{k}},\;n_{iju}^{k}\omega_{i}=\bar{n_{iju}^{k}},\;n_{ijl}^{k}\omega_{j}=\bar{\bar{n_{ijl}^{k}}},\;n_{iju}^{k}\omega_{j}=\bar{\bar{n_{iju}^{k}}}\\ p_{ijl}^{k}\omega_{i}=\bar{p_{ijl}^{k}},\;p_{iju}^{k}\omega_{i}=\bar{p_{iju}^{k}},\;p_{ijl}^{k}\omega_{j}=\bar{\bar{p_{ijl}^{k}}},\;p_{iju}^{k}\omega_{j}=\bar{\bar{p_{iju}^{k}}}\\ r_{ijl}^{*}\omega_{i}=\bar{r_{ijl}^{*}},\;r_{iju}^{*}\omega_{i}=\bar{r_{iju}^{*}},\;r_{ijl}^{*}\omega_{j}=\bar{\bar{r_{ijl}^{*}}},\;r_{iju}^{*}\omega_{j}=\bar{\bar{r_{iju}^{*}}}. \end{array}$$

where $0\leq r_{ijl}^{*},r_{iju}^{*}\leq 1$, so it can be inferred that $\bar{r_{iju}^{*}}\leq \omega_{i}$, and $\bar{\bar{r_{iju}^{*}}}\leq \omega_{j}$. The equivalent linear format of Model (14) is then:

(15) $$\begin{array}{c} \begin{array}{ccc} \;Min\; & & \sum_{k=1}^{m}\sum_{i=1}^{n}\sum_{j=1}^{n}\hat{\omega_{k}}\left(n_{ijl}^{k}+p_{ijl}^{k}\right)+\sum_{k=1}^{m}\sum_{i=1}^{n}\sum_{j=1}^{n}\hat{\omega_{k}}\left(n_{iju}^{k}+p_{iju}^{k}\right)\\ & & s.t.\left\{\begin{array}{cc} r_{ijl}^{k}\omega_{i}-\bar{r_{ijl}^{*}}=\bar{n_{ijl}^{k}}-\bar{p_{ijl}^{k}} & \left(15a\right)\\ r_{ijl}^{k}\omega_{j}-\bar{\bar{r_{ijl}^{*}}}=\bar{\bar{n_{ijl}^{k}}}-\bar{\bar{p_{ijl}^{k}}} & \left(15b\right)\\ r_{iju}^{k}\omega_{i}-\bar{r_{iju}^{*}}=\bar{n_{iju}^{k}}-\bar{p_{iju}^{k}} & \left(15c\right)\\ r_{iju}^{k}\omega_{j}-\bar{\bar{r_{iju}^{*}}}=\bar{\bar{n_{iju}^{k}}}-\bar{\bar{p_{iju}^{k}}} & \left(15d\right)\\ \bar{r_{ijl}^{*}}+\bar{\bar{r_{ijl}^{*}}}\leq \omega_{j}\leq \bar{r_{iju}^{*}}+\bar{\bar{r_{iju}^{*}}} & \left(15e\right)\\ \sum_{i=1}^{n}\omega_{i}=1,0\leq \omega_{i}\leq 1 & \left(15f\right)\\ \bar{r_{iju}^{*}}\leq \omega_{i} & \left(15g\right)\\ \bar{\bar{r_{iju}^{*}}}\leq \omega_{j}\\ 0\leq \bar{n_{ijl}^{k}}\leq n_{ijl}^{k} & \left(15h\right)\\ 0\leq \bar{\bar{n_{ijl}^{k}}}\leq n_{ijl}^{k}\\ 0\leq \bar{n_{iju}^{k}}\leq n_{iju}^{k}\\ 0\leq \bar{\bar{n_{iju}^{k}}}\leq n_{iju}^{k}\\ 0\leq \bar{p_{ijl}^{k}}\leq p_{ijl}^{k}\\ 0\leq \bar{\bar{p_{ijl}^{k}}}\leq p_{ijl}^{k}\\ 0\leq \bar{p_{iju}^{k}}\leq p_{iju}^{k}\\ 0\leq \bar{\bar{p_{iju}^{k}}}\leq p_{iju}^{k}\\ i,j\in N,k\in M & \left(15i\right) \end{array}\right. \end{array} \end{array}$$

where $n_{ijl}^{k}+p_{ijl}^{k}=\left|r_{ijl}^{k}-r_{ijl}^{*}\right|$, $n_{ijl}^{k}-p_{ijl}^{k}=r_{ijl}^{k}-r_{ijl}^{*}$; $n_{iju}^{k}+p_{iju}^{k}=\left|r_{iju}^{k}-r_{iju}^{*}\right|$, and $n_{iju}^{k}-p_{iju}^{k}=r_{iju}^{k}-r_{iju}^{*}$. Formula (15e) is the consistency constraint, indicating that the preference relation for $d^{*}$ satisfies consistency Condition (5).

The objective function of Model (15) indicates that the model can minimize the consensus deviation between judgments $r_{ij}^{*}$ and $r_{ij}^{k}$. For the freedom principle, the optimal (ideal) consensus with appropriate consistency is achieved using Model (15).

### 5.2. Construction of Ideal Fuzzy Preference Relations with II-Consistency Using the Fraternity Principle

Similar to Section 4.2, when p=∞, *U* is the maximum consensus deviation. We let $D_{1}=max_{k,i,j}\hat{\omega_{k}}\left|r_{ijl}^{k}-r_{ijl}^{*}\right|$ and $D_{2}=max_{k,i,j}\hat{\omega_{k}}\left|r_{iju}^{k}-r_{iju}^{*}\right|$ represent the lower and upper deviation limits, respectively, and $min(D_{1}+D_{2})$ represent the minimum sum of the maximum deviation. The optimal (ideal) preference relations using the fraternity principle is shown in Model (16).

(16) $$\begin{array}{c} Min\;(D_{1}+D_{2})\\ s.t.\left\{\begin{array}{cc} r_{ijl}^{k}\omega_{i}-\bar{r_{ijl}^{*}}=\bar{n_{ijl}^{k}}-\bar{p_{ijl}^{k}} & \left(16a\right)\\ r_{ijl}^{k}\omega_{j}-\bar{\bar{r_{ijl}^{*}}}=\bar{\bar{n_{ijl}^{k}}}-\bar{\bar{p_{ijl}^{k}}} & \left(16b\right)\\ r_{iju}^{k}\omega_{i}-\bar{r_{iju}^{*}}=\bar{n_{iju}^{k}}-\bar{p_{iju}^{k}} & \left(16c\right)\\ r_{iju}^{k}\omega_{j}-\bar{\bar{r_{iju}^{*}}}=\bar{\bar{n_{iju}^{k}}}-\bar{\bar{p_{iju}^{k}}} & \left(16d\right)\\ \bar{r_{ijl}^{*}}+\bar{\bar{r_{ijl}^{*}}}\leq \omega_{j}\leq \bar{r_{iju}^{*}}+\bar{\bar{r_{iju}^{*}}} & \left(16e\right)\\ \sum_{i=1}^{n}\omega_{i}=1 & \left(16f\right)\\ \hat{\omega_{k}}\sum_{i=1}^{n}\sum_{j=1}^{n}\left(n_{ijl}^{k}+p_{ijl}^{k}\right)-D_{1}\leq 0 & \left(16g\right)\\ \hat{\omega_{k}}\sum_{i=1}^{n}\sum_{j=1}^{n}\left(n_{iju}^{k}+p_{iju}^{k}\right)-D_{2}\leq 0 & \left(16h\right)\\ \bar{r_{iju}^{*}}\leq \omega_{i} & \left(16i\right)\\ \bar{\bar{r_{iju}^{*}}}\leq \omega_{j}\\ 0\leq \bar{n_{ijl}^{k}}\leq n_{ijl}^{k} & \left(16j\right)\\ 0\leq \bar{\bar{n_{ijl}^{k}}}\leq n_{ijl}^{k}\\ 0\leq \bar{n_{iju}^{k}}\leq n_{iju}^{k}\\ 0\leq \bar{\bar{n_{iju}^{k}}}\leq n_{iju}^{k}\\ 0\leq \bar{p_{ijl}^{k}}\leq p_{ijl}^{k}\\ 0\leq \bar{\bar{p_{ijl}^{k}}}\leq p_{ijl}^{k}\\ 0\leq \bar{p_{iju}^{k}}\leq p_{iju}^{k}\\ 0\leq \bar{\bar{p_{iju}^{k}}}\leq p_{iju}^{k}\\ i,j\in N,k\in M & \left(16k\right) \end{array}\right. \end{array}$$

The parameters used in Model (16) have the same definitions as the parameters used in Model (15). Formulas (16g) and (16h) imply that the lower and upper limits are no more than $D_{1}$ and $D_{2}$, respectively.

The objective function of Model (16) indicates that the model can minimize the highest consensus deviation between judgments $r_{ij}^{*}$ and $r_{ij}^{k}$. For the fraternity principle, the optimal (ideal) consensus with appropriate consistency is achieved using Model (16).

### 5.3. Construction of Ideal Fuzzy Preference Relations with II-Consistency Using the Equality Principle

We let:

(17) $$\begin{array}{c} \hat{\omega_{1}}|r_{ijl}^{1}-r_{ijl}^{*}|=\hat{\omega_{2}}|r_{ijl}^{2}-r_{ijl}^{*}|=\ldots =\hat{\omega_{m}}\left|r_{ijl}^{m}-r_{ijl}^{*}\right|\\ \hat{\omega_{1}}|r_{iju}^{1}-r_{iju}^{*}|=\hat{\omega_{2}}|r_{iju}^{2}-r_{iju}^{*}|=\ldots =\hat{\omega_{m}}\left|r_{iju}^{m}-r_{iju}^{*}\right| \end{array}$$

Equation (17) shows that, in the group decision, the weights for the deviations between the ideal judgment lower limit and the lower limits of the different judgments are equal, and the weights for the deviations between the ideal judgment upper limit and the upper limits of the different judgments are equal. The system function below is used to ensure that Equation (17) is valid.

ωk^(nijlk+pijlk)=ωt^(nijlt+pijlt)ωk^(nijuk+pijuk)=ωt^(nijut+pijut)(18)k=1,…,m−1,t=k+1,…,m.

The optimal ideal preference relation for the equality principle is determined using Model (19). The parameters used in Model (19) have the same definitions as the parameters used in Model (15).

Equations (19g) and (19h) indicate that the weights for the deviations between the ideal DM and each DM are as equal as possible. $\eta_{ij}^{kt}$, $\rho_{ij}^{kt}$ are the positive and negative deviations of $\hat{\omega_{k}}\left(n_{ijl}^{k}+p_{ijl}^{k}\right)$ and $\hat{\omega_{t}}\left(n_{ijl}^{t}+p_{ijl}^{t}\right)$, respectively, and $\eta_{ij}^{\prime kt}$, $\rho_{ij}^{\prime kt}$ are the positive and negative deviations of $\hat{\omega_{k}}\left(n_{iju}^{k}+p_{iju}^{k}\right)$ and $\hat{\omega_{k}}\left(n_{iju}^{t}+p_{iju}^{t}\right)$, respectively. Clearly, $\left(\eta_{ij}^{kt}+\rho_{ij}^{kt}\right)$ and $\left(\eta_{ij}^{\prime kt}+\rho_{ij}^{\prime kt}\right)$ in the objective function should be as small as possible.

The objective function of Model (19) indicates that the model can ensure that the weights for deviations between the ideal judgment $r_{ij}^{*}$ and the judgments made by the DMs are as equal as possible. For the equality principle, the optimal (ideal) consensus with appropriate consistency is achieved using Model (19).

(19) $$\begin{array}{c} \begin{array}{cc} Min\;\sum_{k=1}^{m-1}\sum_{t=k+1}^{m}\left(\eta_{ij}^{kt}+\rho_{ij}^{kt}\right)+\sum_{k=1}^{m-1}\sum_{t=k+1}^{m}\left(\eta_{ij}^{\prime kt}+\rho_{ij}^{\prime kt}\right) & \\ s.t.\left\{\begin{array}{cc} r_{ijl}^{k}\omega_{i}-\bar{r_{ijl}^{*}}=\bar{n_{ijl}^{k}}-\bar{p_{ijl}^{k}} & \left(19a\right)\\ r_{ijl}^{k}\omega_{j}-\bar{\bar{r_{ijl}^{*}}}=\bar{\bar{n_{ijl}^{k}}}-\bar{\bar{p_{ijl}^{k}}} & \left(19b\right)\\ r_{iju}^{k}\omega_{i}-\bar{r_{iju}^{*}}=\bar{n_{iju}^{k}}-\bar{p_{iju}^{k}} & \left(19c\right)\\ r_{iju}^{k}\omega_{j}-\bar{\bar{r_{iju}^{*}}}=\bar{\bar{n_{iju}^{k}}}-\bar{\bar{p_{iju}^{k}}} & \left(19d\right)\\ \bar{r_{ijl}^{*}}+\bar{\bar{r_{ijl}^{*}}}\leq \omega_{j}\leq \bar{r_{iju}^{*}}+\bar{\bar{r_{iju}^{*}}} & \left(19e\right)\\ \sum_{i=1}^{n}\omega_{i}=1 & \left(19f\right)\\ \hat{\omega_{k}}\left(n_{ijl}^{k}+p_{ijl}^{k}\right)-\hat{\omega_{t}}\left(n_{ijl}^{t}+p_{ijl}^{t}\right)\\ +\eta_{ij}^{kt}-\rho_{ij}^{kt}=0 & \left(19g\right)\\ \hat{\omega_{k}}\left(n_{iju}^{k}+p_{iju}^{k}\right)-\hat{\omega_{t}}\left(n_{iju}^{t}+p_{iju}^{t}\right)\\ +\eta_{ij}^{\prime kt}-\rho_{ij}^{\prime kt}=0 & \left(19h\right)\\ k=1,...,m-1,t=k+1,...,m\\ \bar{r_{iju}^{*}}\leq \omega_{i} & \left(19i\right)\\ \bar{\bar{r_{iju}^{*}}}\leq \omega_{j}\\ 0\leq \bar{n_{ijl}^{k}}\leq n_{ijl}^{k} & \left(19j\right)\\ 0\leq \bar{\bar{n_{ijl}^{k}}}\leq n_{ijl}^{k}\\ 0\leq \bar{n_{iju}^{k}}\leq n_{iju}^{k}\\ 0\leq \bar{\bar{n_{iju}^{k}}}\leq n_{iju}^{k}\\ 0\leq \bar{p_{ijl}^{k}}\leq p_{ijl}^{k}\\ 0\leq \bar{\bar{p_{ijl}^{k}}}\leq p_{ijl}^{k}\\ 0\leq \bar{p_{iju}^{k}}\leq p_{iju}^{k}\\ 0\leq \bar{\bar{p_{iju}^{k}}}\leq p_{iju}^{k}\\ i,j\in N,k\in M & \left(19k\right) \end{array}\right. \end{array} \end{array}$$

### 5.4. Construction of Ideal Fuzzy Preference Relations with II-Consistency Using Mixed Principles

Similar to Section 4.4, integrating Models (15) and (16) with Model (19) introduces the social pattern weights $\lambda_{i}$, $0\leq \lambda_{i}\leq 1$, i=1,2. The optimal ideal preference relation is shown in Model (20).

(20) $$\begin{array}{c} \begin{array}{ccc} Min & & \lambda_{1}\left(D_{1}+D_{2}\right)+\lambda_{2}\left[\sum_{k=1}^{m}\sum_{i=1}^{n}\sum_{j=1}^{n}\hat{\omega_{k}}\left(n_{ijl}^{k}+p_{ijl}^{k}\right)+\sum_{k=1}^{m}\sum_{i=1}^{n}\sum_{j=1}^{n}\hat{\omega_{k}}\left(n_{iju}^{k}+p_{iju}^{k}\right)\right]\\ & & +\left(1-\lambda_{1}-\lambda_{2}\right)\left[\sum_{k=1}^{m-1}\sum_{t=k+1}^{m}\left(\eta_{kt}+\rho_{kt}\right)+\sum_{k=1}^{m-1}\sum_{t=k+1}^{m}\left(\eta_{kt}^{\prime }+\rho_{kt}^{\prime }\right)\right]\\ & & s.t.\left\{\begin{array}{cc} r_{ijl}^{k}\omega_{i}-\bar{r_{ijl}^{*}}=\bar{n_{ijl}^{k}}-\bar{p_{ijl}^{k}} & \left(20a\right)\\ r_{ijl}^{k}\omega_{j}-\bar{\bar{r_{ijl}^{*}}}=\bar{\bar{n_{ijl}^{k}}}-\bar{\bar{p_{ijl}^{k}}} & \left(20b\right)\\ r_{iju}^{k}\omega_{i}-\bar{r_{iju}^{*}}=\bar{n_{iju}^{k}}-\bar{p_{iju}^{k}} & \left(20c\right)\\ r_{iju}^{k}\omega_{j}-\bar{\bar{r_{iju}^{*}}}=\bar{\bar{n_{iju}^{k}}}-\bar{\bar{p_{iju}^{k}}} & \left(20d\right)\\ \bar{r_{ijl}^{*}}+\bar{\bar{r_{ijl}^{*}}}\leq \omega_{j}\leq \bar{r_{iju}^{*}}+\bar{\bar{r_{iju}^{*}}} & \left(20e\right)\\ \sum_{i=1}^{n}\omega_{i}=1 & \left(20f\right)\\ \hat{\omega_{k}}\sum_{i=1}^{n}\sum_{j=1}^{n}\left(n_{ijl}^{k}+p_{ijl}^{k}\right)-D_{1}\leq 0 & \left(20g\right)\\ \hat{\omega_{k}}\sum_{i=1}^{n}\sum_{j=1}^{n}\left(n_{iju}^{k}+p_{iju}^{k}\right)-D_{2}\leq 0 & \left(20h\right)\\ \hat{\omega_{k}}\left(n_{ijl}^{k}+p_{ijl}^{k}\right)-\hat{\omega_{t}}\left(n_{ijl}^{t}+p_{ijl}^{t}\right) & \\ +\eta_{kt}-\rho_{kt}=0 & \left(20i\right)\\ \hat{\omega_{k}}\left(n_{iju}^{k}+p_{iju}^{k}\right)-\hat{\omega_{t}}\left(n_{iju}^{t}+p_{iju}^{t}\right) & \\ +\eta_{kt}^{\prime }-\rho_{kt}^{\prime }=0 & \left(20j\right)\\ k=1,...,m-1,t=k+1,...,m\\ \bar{r_{iju}^{*}}\leq \omega_{i} & \left(20k\right)\\ \bar{\bar{r_{iju}^{*}}}\leq \omega_{j}\\ 0\leq \bar{n_{ijl}^{k}}\leq n_{ijl}^{k} & \left(20l\right)\\ 0\leq \bar{\bar{n_{ijl}^{k}}}\leq n_{ijl}^{k}\\ 0\leq \bar{n_{iju}^{k}}\leq n_{iju}^{k}\\ 0\leq \bar{\bar{n_{iju}^{k}}}\leq n_{iju}^{k}\\ 0\leq \bar{p_{ijl}^{k}}\leq p_{ijl}^{k}\\ 0\leq \bar{\bar{p_{ijl}^{k}}}\leq p_{ijl}^{k}\\ 0\leq \bar{p_{iju}^{k}}\leq p_{iju}^{k}\\ 0\leq \bar{\bar{p_{iju}^{k}}}\leq p_{iju}^{k}\\ i,j\in N,k\in M & \left(20m\right) \end{array}\right. \end{array} \end{array}$$

The parameters used in Model (20) have the same definitions as the parameters used in the three models described above. $\lambda_{1}$, $\lambda_{2}$ and $\left(1-\lambda_{1}-\lambda_{2}\right)$ are determined for the decision-making process and control the weights of the freedom, fraternity and equality principles, respectively.

The objective function of Model (20) indicates that the model can minimize the consensus deviation between $r_{ij}^{*}$ and $r_{ij}^{k}$. For mixed principles, the optimal (ideal) consensus with appropriate consistency is achieved using Model (20).

## 6. Case Study of a Method for Selecting a Cooking Method to Decrease the POP Concentrations in Food

The cooking method used can affect the POP contents and distributions in food. We will only consider frying, baking, poaching and pressure cooking ($r_{i}\left(i=1,2,3,4\right)$, respectively) and use grass carp (a freshwater fish) as a model food of animal origin. Three experts, $\left(R^{i},i=1,2,3\right)$, provide interval fuzzy preference relations, then we will determine the ideal cooking method using the four social principles mentioned above and summarized below.

Freedom principle: The total disparities between the expert and ideal advice are minimized without ignoring the opinion of any expert.

Fraternity principle: The maximum disparities between the expert and ideal advice are minimized.

Equality principle: The advice of each expert is used equally.

Mixed principle: The three social principles described above are all used.

The $R^{1}-R^{3}$ arrays are shown below.

$$\begin{array}{c} \begin{array}{c} R^{1}={\left(r_{ij}^{1}\right)}_{4\times 4}=\left(\begin{array}{cccc} \left[0.5,0.5\right] & \left[0.2,0.4\right] & \left[0.3,0.4\right] & \left[0.6,0.9\right]\\ \left[0.6,0.8\right] & \left[0.5,0.5\right] & \left[0.6,0.7\right] & \left[0.7,0.9\right]\\ \left[0.6,0.7\right] & \left[0.3,0.4\right] & \left[0.5,0.5\right] & \left[0.7,0.8\right]\\ \left[0.1,0.4\right] & \left[0.1,0.3\right] & \left[0.2,0.3\right] & \left[0.5,0.5\right] \end{array}\right) \end{array}. \end{array}$$

$$\begin{array}{c} \begin{array}{c} R^{2}={\left(r_{ij}^{2}\right)}_{4\times 4}=\left(\begin{array}{cccc} \left[0.5,0.5\right] & \left[0.1,0.3\right] & \left[0.5,0.6\right] & \left[0.7,0.8\right]\\ \left[0.7,0.9\right] & \left[0.5,0.5\right] & \left[0.5,0.9\right] & \left[0.6,0.8\right]\\ \left[0.1,0.5\right] & \left[0.3,0.5\right] & \left[0.5,0.5\right] & \left[0.5,0.7\right]\\ \left[0.2,0.3\right] & \left[0.2,0.4\right] & \left[0.3,0.5\right] & \left[0.5,0.5\right] \end{array}\right) \end{array}. \end{array}$$

$$\begin{array}{c} \begin{array}{c} R^{3}={\left(r_{ij}^{3}\right)}_{4\times 4}=\left(\begin{array}{cccc} \left[0.5,0.5\right] & \left[0.3,0.4\right] & \left[0.1,0.4\right] & \left[0.2,0.4\right]\\ \left[0.6,0.7\right] & \left[0.5,0.5\right] & \left[0.6,0.8\right] & \left[0.5,0.8\right]\\ \left[0.6,0.9\right] & \left[0.2,0.4\right] & \left[0.5,0.5\right] & \left[0.6,0.9\right]\\ \left[0.6,0.8\right] & \left[0.2,0.5\right] & \left[0.1,0.4\right] & \left[0.5,0.5\right] \end{array}\right) \end{array}. \end{array}$$

The weights of the three experts are $\hat{\omega_{1}}=0.1$, $\hat{\omega_{2}}=0.3$ and $\hat{\omega_{3}}=0.6$. The weight is the proportion of an expert’s opinion used when determining the priority of a particular cooking method. For the mixed principle, $\lambda_{1}=0.2$ is the weight of the freedom principle, $\lambda_{2}=0.3$ is the weight of the fraternity principle and $\left(1-\lambda_{1}-\lambda_{2}\right)$ is the weight of the equality principle.

We will now assess the priorities of the different cooking methods with *I*-consistency and II-consistency using the freedom, fraternity, equality and mixed principles.

### 6.1. I-Consistency

The optimal solutions to Models (7), (8), (11) and (12) are shown in Table 1. Here, we will analyze the results for the freedom principle as an example. The priority weights for the freedom, fraternity, equality and mixed principles are 0.1768, 0.4188, 0.2573 and 0.1471, respectively, and the priority relations for the four cooking methods are $r_{2}≻r_{3}≻r_{1}≻r_{4}$. This demonstrates that, using the freedom principle, baking decreases the POP contents of grass carp most, poaching next, frying next and pressure cooking least. That is to say, under the conditions described, the first choice for decreasing the POP contents of grass carp is to bake the fish.

Explanations of the priorities found using the other principles are omitted.

The ideal preference relations using all of the social principles are shown below.

The ideal preference relations using the freedom principle are:

$$\begin{array}{c} \begin{array}{c} R^{*}=\left(\begin{array}{cccc} 1 & 0.2958 & 0.4114 & 0.5414\\ 0.7042 & 1 & 0.6156 & 0.7456\\ 0.5886 & 0.3844 & 1 & 0.63\\ 0.4586 & 0.2544 & 0.37 & 1 \end{array}\right) \end{array}. \end{array}$$

The ideal preference relations using the fraternity principle are:

$$\begin{array}{c} \begin{array}{c} R^{*}=\left(\begin{array}{cccc} 1 & 0.2865 & 0.3995 & 0.5274\\ 0.7135 & 1 & 0.6130 & 0.7409\\ 0.6005 & 0.387 & 1 & 0.6279\\ 0.4726 & 0.2591 & 0.3721 & 1 \end{array}\right) \end{array}. \end{array}$$

The ideal preference relations using the equality principle are:

$$\begin{array}{c} \begin{array}{c} R^{*}=\left(\begin{array}{cccc} 1 & 0.3406 & 0.4203 & 0.5204\\ 0.6594 & 1 & 0.5796 & 0.6797\\ 0.5797 & 0.4204 & 1 & 0.6001\\ 0.4796 & 0.3103 & 0.3999 & 1 \end{array}\right) \end{array}. \end{array}$$

The ideal preference relations using the mixed principle are:

$$\begin{array}{c} \begin{array}{c} R^{*}=\left(\begin{array}{cccc} 1 & 0.3067 & 0.4367 & 0.5033\\ 0.6933 & 1 & 0.6258 & 0.7259\\ 0.5633 & 0.3742 & 1 & 0.6140\\ 0.4967 & 0.2741 & 0.386 & 1 \end{array}\right) \end{array}. \end{array}$$

### 6.2. II-Consistency

The optimal solutions to Models (15), (16), (19) and (20) are shown in Table 2. Here, we will analyze the results for the freedom principle as an example. The priority weights for the freedom, fraternity, equality and mixed principles are 0.1714, 0.4, 0.2571 and 0.1714, respectively, and the priority relations for the four cooking methods are $r_{2}≻r_{3}≻r_{1}=r_{4}$. This demonstrates that, using the freedom principle, baking decreases the POP contents of grass carp most, poaching next and frying and pressure cooking both least. That is to say, under the conditions described, the first choice for decreasing the POP contents of grass carp is to bake the fish.

Explanations of the priorities found using the other principles are omitted.

The ideal preference relations using all of the social principles are shown below.

The ideal preference relations using the freedom principle are:

$$\begin{array}{c} \begin{array}{c} R^{*}=\left(\begin{array}{cccc} \left[0.5,0.5\right] & \left[0.2999,0.4002\right] & \left[0.0998,0.4002\right] & \left[0.2001,0.5\right]\\ \left[0.5998,0.7001\right] & \left[0.5,0.5\right] & \left[0.6,0.8\right] & \left[0.5,0.8\right]\\ \left[0.5998,0.9002\right] & \left[0.2,0.4\right] & \left[0.5,0.5\right] & \left[0.6002,0.9\right]\\ \left[0.5,0.7999\right] & \left[0.2,0.5\right] & \left[0.1,0.3998\right] & \left[0.5,0.5\right] \end{array}\right) \end{array}. \end{array}$$

The ideal preference relations using the fraternity principle are:

$$\begin{array}{c} \begin{array}{c} R^{*}=\left(\begin{array}{cccc} \left[0.5,0.5\right] & \left[0.2399,0.3843\right] & \left[0.2184,0.5147\right] & \left[0.3343,0.5642\right]\\ \left[0.6157,0.7601\right] & \left[0.5,0.5\right] & \left[0.5692,0.8201\right] & \left[0.5399,0.8001\right]\\ \left[0.4853,0.7816\right] & \left[0.1799,0.4308\right] & \left[0.5,0.5\right] & \left[0.4998,0.8541\right]\\ \left[0.4358,0.6657\right] & \left[0.1999,0.4601\right] & \left[0.1459,0.5002\right] & \left[0.5,0.5\right] \end{array}\right) \end{array}. \end{array}$$

The ideal preference relations using the equality principle are:

$$\begin{array}{c} \begin{array}{c} R^{*}=\left(\begin{array}{cccc} \left[0.5,0.5\right] & \left[0.3142,0.6785\right] & \left[0.3271,0.7195\right] & \left[0.3891,0.7781\right]\\ \left[0.3215,0.6858\right] & \left[0.5,0.5\right] & \left[0.4445,0.8246\right] & \left[0.4618,0.8398\right]\\ \left[0.2805,0.6729\right] & \left[0.1754,0.5555\right] & \left[0.5,0.5\right] & \left[0.4433,0.8355\right]\\ \left[0.2219,0.6109\right] & \left[0.1602,0.5382\right] & \left[0.1645,0.5567\right] & \left[0.5,0.5\right] \end{array}\right) \end{array}. \end{array}$$

The ideal preference relations using the mixed principle are:

$$\begin{array}{c} \begin{array}{c} R^{*}=\left(\begin{array}{cccc} \left[0.5,0.5\right] & \left[0.2395,0.3946\right] & \left[0.1975,0.4657\right] & \left[0.3664,0.5333\right]\\ \left[0.6054,0.7605\right] & \left[0.5,0.5\right] & \left[0.5668,0.8333\right] & \left[0.5333,0.8145\right]\\ \left[0.5343,0.8025\right] & \left[0.1667,0.4332\right] & \left[0.5,0.5\right] & \left[0.5667,0.8333\right]\\ \left[0.4667,0.6336\right] & \left[0.1855,0.4667\right] & \left[0.1667,0.4333\right] & \left[0.5,0.5\right] \end{array}\right) \end{array}. \end{array}$$

The results for the two consistency conditions were compared to support our conclusions. Some data in the matrices were changed (changing one matrix each time) to seek to identify a pattern. The results for the two types of consistency with the modified matrices are shown in Table 3 (for *I*-consistency) and Table 4 (for II-consistency). It can be seen that, using the same social principle, the same weights were always found using II-consistency, making it difficult to identify the priority cooking method. The weights obtained using *I*-consistency were all different. Therefore, even though more experts suggest using II-consistency than *I*-consistency, we found that *I*-consistency is more acceptable and robust.

## 7. Conclusions

Improving the POP contents and distributions in food of animal origin can protect human health. Different cooking methods should be selected to decrease POP concentrations in food, which depends on the environment and the food type. We investigated the CDM problem using interval preference relations and two consistency definitions. We used the goal programming approach to minimize the distances between ideal judgments and the judgments of individual experts and developed the ideal interval preference relation models to prioritize different cooking methods. A numerical example indicated that *I*-consistency is more appropriate than II-consistency for this problem. The best cooking method to decrease POP concentrations in a particular food (grass carp) was identified by using the simulation method.

(1) In the first model class, based on freedom, fraternity, equality and mixed principles, we considered deviations between the ideal judgment and the judgments of individual experts and achieved the minimum deviation in consistency.

(2) In the second model class, also based on freedom, fraternity, equality and mixed principles, the goal was to achieve the minimum deviation between the ideal expected judgment and the judgment of each individual expert. Consistency was used as a constraint.

The results of the numerical example indicated that *I*-consistency is more appropriate than II-consistency.

This research could be extended to consensus decision-making models based on triangular fuzzy preference relations, intuitionistic fuzzy preference relations, hesitant fuzzy preference relations, linguistic preference relations and other relations. The model can take the views of different experts expressed in different ways into consideration, making it appropriate for use with complicated ecological systems and cooking methods to allow cooking methods to be prioritized to minimize human exposure to POPs.

## Figures and Tables

**Table 1:** Results of the model with *I*-consistency.

	Freedom	Fraternity	Equality	Mixture
min	0.2010	0.096	0	0.0481
*D*		0.096		0.0956
$\omega_{1}$	0.1768	0.1699	0.1909	0.1805
$\omega_{2}$	0.4188	0.4194	0.3704	0.4136
$\omega_{3}$	0.2573	0.2603	0.2653	0.2453
$\omega_{4}$	0.1471	0.1504	0.1734	0.1605
$\alpha_{12}^{1}$	0.4792	0.5262	0.5917	0.5274
$\alpha_{13}^{1}$	1	0.6243	0.5697	0.6237
$\alpha_{14}^{1}$	0.1	0.2549	0.4276	0.4099
$\alpha_{23}^{1}$	0.1559	0.4842	0.5285	0.2584
$\alpha_{24}^{1}$	0.2279	0.3931	0.4644	0.1297
$\alpha_{34}^{1}$	0.1	0.4034	0.5105	0.1
$\alpha_{12}^{2}$	0.9792	0.9361	0.6361	1
$\alpha_{13}^{2}$	0.1	0.1123	0.5288	0.1
$\alpha_{14}^{2}$	0.1	0.1112	0.4961	0.1
$\alpha_{23}^{2}$	0.2890	0.2815	0.4384	0.3146
$\alpha_{24}^{2}$	0.7279	0.7058	0.5026	0.6297
$\alpha_{34}^{2}$	0.65	0.6404	0.5273	0.5564
$\alpha_{12}^{3}$	0.1	0.1	0.5573	0.1
$\alpha_{13}^{3}$	1	0.9982	0.6737	1
$\alpha_{14}^{3}$	1	1	0.6842	1
$\alpha_{23}^{3}$	0.1	0.1	0.4649	0.1292
$\alpha_{24}^{3}$	0.8186	0.8030	0.5197	0.7531
$\alpha_{34}^{3}$	0.1	0.1	0.4081	0.1

**Table 2:** Results of the model with II-consistency.

	min	$\omega_{1}$	$\omega_{2}$	$\omega_{3}$	$\omega_{4}$
freedomprinciple	0.2369	0.1714	0.4	0.2571	0.1714
fraternityprinciple	0.12	0.2001	0.3997	0.2001	0.2001
equalityprinciple	0	0.2339	0.2891	0.2547	0.2222
mixedprinciples	0.1137	0.2025	0.3347	0.2603	0.2025

**Table 3:** Priorities determined using the modified matrices and *I*-consistency.

Principle	Weight	$\mathrm{Change}R^{1}$	$\mathrm{Change}R^{2}$	$\mathrm{Change}R^{3}$
freedom	$\omega_{1}$	0.1780	0.1698	0.1803
	$\omega_{2}$	0.4135	0.3731	0.3920
	$\omega_{3}$	0.2596	0.2573	0.2414
	$\omega_{4}$	0.1488	0.1998	0.1858
fraternity	$\omega_{1}$	0.1709	0.1623	0.1868
	$\omega_{2}$	0.4169	0.3520	0.4177
	$\omega_{3}$	0.2618	0.2347	0.2397
	$\omega_{4}$	0.1504	0.2510	0.1555
eequality	$\omega_{1}$	0.2008	0.2053	0.2271
	$\omega_{2}$	0.3505	0.3201	0.3604
	$\omega_{3}$	0.2545	0.2740	0.2460
	$\omega_{4}$	0.1942	0.2005	0.1665
mixture	$\omega_{1}$	0.1804	0.1616	0.2023
	$\omega_{2}$	0.4055	0.4045	0.4121
	$\omega_{3}$	0.2447	0.2517	0.2388
	$\omega_{4}$	0.1693	0.1820	0.1439

**Table 4:** Priorities determined using the modified matrices and II-consistency.

Principle	Weight	$\mathrm{Change}R^{1}$	$\mathrm{Change}R^{2}$	$\mathrm{Change}R^{3}$
freedom	$\omega_{1}$	0.1875	0.25	0.25
	$\omega_{2}$	0.4375	0.25	0.25
	$\omega_{3}$	0.1875	0.25	0.25
	$\omega_{4}$	0.1875	0.25	0.25
fraternity	$\omega_{1}$	0.2037	0.25	0.25
	$\omega_{2}$	0.3899	0.25	0.25
	$\omega_{3}$	0.2037	0.25	0.25
	$\omega_{4}$	0.2037	0.25	0.25
equality	$\omega_{1}$	0.2364	0.2465	0.2550
	$\omega_{2}$	0.2891	0.2725	0.2790
	$\omega_{3}$	0.2504	0.2524	0.2443
	$\omega_{4}$	0.2241	0.2286	0.2216
mixture	$\omega_{1}$	0.2118	0.2286	0.2283
	$\omega_{2}$	0.3294	0.2571	0.3302
	$\omega_{3}$	0.2471	0.2571	0.2568
	$\omega_{4}$	0.2118	0.2571	0.1848

## References

[1] Jaacks L.M., Boyd Barr D., Sundaram R., Grewal J., Zhang C., Buck Louis G.M. (2016). Pre-Pregnancy Maternal Exposure to Persistent Organic Pollutants and Gestational Weight Gain: A Prospective Cohort Study. Int. J. Environ. Res. Public Health.

[2] Lee H.A., Su H.P., Hong Y.S., Ha E.H., Park H. (2016). The Effect of Exposure to Persistent Organic Pollutants on Metabolic Health among KOREAN Children during a 1-Year Follow-Up. Int. J. Environ. Res. Public Health.

[3] Zhou P., Zhao Y., Li J., Wu G., Zhang L., Liu Q., Fan S., Yang X., Li X., Wu Y. (2012). Dietary exposure to persistent organochlorine pesticides in 2007 Chinese total diet study. Environ. Int..

[4] Domingo J.L. (2010). Influence of cooking processes on the concentrations of toxic metals and various organic environmental pollutants in food: A review of the published literature. Crit. Rev. Food Sci. Nutr..

[5] Rawn D.F., Breakell K., Verigin V., Tittlemier S.A., Del Gobbo L., Diamond M., Vanderlinden L., Sit D. (2013). Impacts of cooking technique on polychlorinated biphenyl and polychlorinated dioxins/furan concentrations in fish and fish products with intake estimates. J. Agric. Food Chem..

[6] Boer J.D., Lammertse N., Koekkoek J., van Hattum B. (2013). PCB and organochlorine pesticide concentrations in eel increase after frying. Chemosphere.

[7] Zhang H. (2016). A goal programming model of obtaining the priority weights from an interval preference relation. Inf. Sci..

[8] Xu Z., Chen J. (2008). Some models for deriving the priority weights from interval fuzzy preference relations. Eur. J. Oper. Res..

[9] Liu F., Zhang W.G., Zhang L.H. (2014). Consistency analysis of triangular fuzzy reciprocal preference relations. Eur. J. Oper. Res..

[10] Wang Z.J., Tong X. (2016). Consistency analysis and group decision making based on triangular fuzzy additive reciprocal preference relations. Inf. Sci. Int. J..

[11] Qian W., Wang Z.J., Li K.W. (2016). Medical Waste Disposal Method Selection Based on a Hierarchical Decision Model with Intuitionistic Fuzzy Relations. Int. J. Environ. Res. Public Health.

[12] Tong X., Wang Z.J. (2016). A Group Decision Framework with Intuitionistic Preference Relations and Its Application to Low Carbon Supplier Selection. Int. J. Environ. Res. Public Health.

[13] Wang J., Lan J., Ren P., Luo Y. (2012). Some programming models to derive priority weights from additive interval fuzzy preference relation. Knowl.-Based Syst..

[14] Hu M., Ren P., Lan J., Zheng W. (2014). Note on “Some models for deriving the priority weights from interval fuzzy preference relations”. Eur. J. Oper. Res..

[15] Chu J., Liu X., Wang Y., Chin K.S. (2016). A group decision making model considering both the additive consistency and consensus of intuitionistic fuzzy preference relations. Comput. Ind. Eng..

[16] Bezdek J., Spillman B., Spillman R. (1978). A fuzzy relation space for group decision theory. Fuzzy Sets Syst..

[17] Orive R. (1988). Consensus, action immediacy, and opinion confidence. Pers. Soc. Psychol. Bull..

[18] Chiclana F., Garcia J.M.T., Moral M.J.D., Herrera-Viedma E. (2013). A statistical comparative study of different similarity measures of consensus in group decision making. Inf. Sci..

[19] Zhang H. (2015). A consistency model for group decision making problems with interval multiplicative preference relations. Appl. Soft Comput..

[20] González-Pachón J., Romero C. (2016). Bentham, Marx and Rawls ethical principles: In search for a compromise. Omega.

[21] Wu J., Chiclana F. (2014). A social network analysis trust-consensus based approach to group decision-making problems with interval-valued fuzzy reciprocal preference relations. Knowl.-Based Syst..

[22] Bentham J. (1961). The Utilitarians: An Introduction to the Principles of Morals and Legislation. Gen. Inf..

[23] Rawls L. (1973). A Theory of Justice.

[24] Wu J. (2004). An Aggregation Method for Group Preference Information of Interval Number Complementary Judgement Matrices. Syst. Eng. Theory Methodol. Appl..

[25] Xu Z.S. (2002). Two Methods for Priorities of Complementary Judgement Matrices—Weighted Least-square Method and Eigenvector Method. Syst. Eng. Theory Pract..

[26] Song X., Li Y. (2003). Approaches to transformation between AHP judgment matrix and fuzzy judgment matrix. J. Dalian Univ. Technol..

[27] Gong Z.W. (2008). Least-square method to priority of the fuzzy preference relations with incomplete information. Int. J. Approx. Reason..

